# A novel experimental approach to characterize neutron fields at high- and low-energy particle accelerators

**DOI:** 10.1038/s41598-022-21113-7

**Published:** 2022-10-07

**Authors:** Saverio Braccini, Pierluigi Casolaro, Gaia Dellepiane, Isidre Mateu, Lorenzo Mercolli, Andrea Pola, Dario Rastelli, Paola Scampoli

**Affiliations:** 1 Albert Einstein Center for Fundamental Physics (AEC), Laboratory for High Energy Physics (LHEP), University of Bern, Sidlerstrasse 5, 3012 Bern, Switzerland; 2 Department of Nuclear Medicine, Inselspital, Bern University Hospital, University of Bern, Freiburgstrasse 18, 3010 Bern, Switzerland; 3 Politecnico di Milano, Dipartimento di Energia, Via La Masa 34, 20156 Milano, Italy; 4 Raylab s.r.l., Via Monte di Pietà 2, 24043 Caravaggio, Bergamo Italy; 5 Department of Physics ”Ettore Pancini”, University of Napoli Federico II, Complesso Universitario di Monte S. Angelo, 80126 Napoli, Italy

**Keywords:** Nuclear physics, Experimental nuclear physics

## Abstract

The characterization of particle accelerator induced neutron fields is challenging but fundamental for research and industrial activities, including radiation protection, neutron metrology, developments of neutron detectors for nuclear and high-energy physics, decommissioning of nuclear facilities, and studies of neutron damage on materials and electronic components. This work reports on the study of a novel approach to the experimental characterization of neutron spectra at two complex accelerator environments, namely the CERF, a high-energy mixed reference field at CERN in Geneva, and the Bern medical cyclotron laboratory, a facility used for multi-disciplinary research activities, and for commercial radioisotope production for nuclear medicine. Measurements were performed through an innovative active neutron spectrometer called DIAMON, a device developed to provide in real time neutron energy spectra without the need of guess distributions. The intercomparison of DIAMON measurements with reference data, Monte Carlo simulations, and with the well-established neutron monitor Berthold LB 6411, has been found to be highly satisfactory in all conditions. It was demonstrated that DIAMON is an almost unique device able to characterize neutron fields induced by hadrons at 120 GeV/c as well as by protons at 18 MeV colliding with different materials. The accurate measurement of neutron spectra at medical cyclotrons during routine radionuclide production for nuclear medicine applications is of paramount importance for the facility decommissioning. The findings of this work are the basis for establishing a methodology for producing controlled proton-induced neutron beams with medical cyclotrons.

## Introduction

The accurate knowledge of the energy spectrum and dose of neutron fields is essential in several areas of fundamental and applied physics, including radiation protection, radionuclide production, decommissioning of nuclear sites and radiation hardness. Generally, accelerators of energy above ~10 MeV produce secondary neutrons, which represent a hazard in areas accessible to the personnel^1^. Furthermore, secondary neutrons produced in proton therapy facilities through the interactions of the primary beam with the patient and with the surrounding instrumentation, lead to an unwanted neutron dose contribution to the patient in tissues outside the target volume^2,3^. Often, neutron spectra are poorly known and the neutron dose cannot be easily assessed. The activation of materials (walls and/or any fixed equipment) in experimental areas surrounding particle beams is mainly caused by secondary neutrons produced during the irradiation. The study of the activation of these materials is necessary for the decommissioning phase of existing and new facilities^4^. Along this line, a considerable scientific effort has been done in the last years in order to characterize secondary neutrons produced at accelerators for the production of nuclear medicine radioisotopes^4–9^. In addition, Jeffries et al. characterized the neutron fluence rate during a production of ^18^F at a medical cyclotron for evaluating its potential use in neutron damage studies^10^. The neutron-induced damage on materials and electronics is a hot topic for space and avionics, as well as for detectors used in medical and High Energy Physics (HEP) applications^11–17^.

Experimentally, neutron spectrometry is challenging. Also the evaluation of the neutron dose in workplaces is challenging as it depends on a quality factor curve which in turn strongly depends on the neutron energy^18^. Neutron monitoring can be performed with rem counters, devices that typically exploit a thermal neutron detector such as BF_3_ or ^3^He proportional counters embedded in moderating materials^19,20^. Rem counters are designed to have a response with an energy dependence as similar as possible to that of the neutron fluence to ambient dose equivalent conversion coefficients $\dot{h}^{*}(10)$ recommended by the International Commission of Radiation Protection (ICRP). A well-established rem counter is the Berthold LB 6411, operating from thermal energies to 20 MeV in the dose rate range from 30 nSv/h to 100 mSv/h^21^. It should be noted that the ICRP conversion coefficients have recently been revised (from $\dot{h}^{*}(10)$ to $\dot{h}^{*}$). Therefore, the outputs of monitors need an upgrade.

Neutron spectrometry techniques are mostly based on Time-Of-Flight (TOF) measurement systems, nuclear reaction and nuclear recoil based spectrometers, and Bonner spheres^22–24^. TOF detection systems measure the neutron TOF by means of one or two neutron detectors, typically scintillation, silicon or gaseous detectors^25–27^. TOF measurements are typically performed in the MeV energy range and are characterized by high energy resolution (of a few percent). However they require complex detection and electronics systems, complex calibration procedures and wide space. Nuclear recoil spectrometers are based on the detection of recoils produced by the neutron-proton scattering typically by means of organic scintillators and proportional counters^22,28,29^. The detection efficiencies are typically low and the recorded pulse height spectrum must be processed through an unfolding algorithm in order to derive the incident neutron spectrum^30^.

Bonner spheres are a set of moderating spheres of different size with one (or more) thermal neutron detector(s) placed in their center. A single Bonner sphere can be used as a neutron monitor, whereas a set of Bonner spheres of different size can be used to measure the neutron spectrum from thermal (0.025 eV) to high (typically 20 MeV or 1 GeV, depending on the type of Bonner spheres used) energies. In particular, Bonner sphere readings are analyzed by means of specific unfolding procedures which relay on a good knowledge of the response functions to monoenergetic neutrons for each sphere. The unfolding procedure, typically based on Monte Carlo methods, compares the measured counting rate with the calculated one, the latter obtained by considering an initial parameter which defines a neutron guess-spectrum^31,32^. The outputs consist in a set of acceptable solutions from which the best is derived with different approaches to provide the final solution.

An innovative neutron spectrometer named DIAMON has recently been developed by the Nuclear Measurement Laboratory of Politecnico di Milano and RAYLAB, a spin-off company of Politecnico di Milano^33^. As explained in the “Methods” section, DIAMON (Direction-aware Isotropic and Active neutron MONitor with spectrometric capabilities) is an all-in-one system featuring a quasi-ideal response as a function of the neutron energy. It provides neutron spectra, directions, and field quantities in real-time by means of a self-running unfolding code, which doesn’t need a guess spectrum. All these features pave the way towards a novel experimental approach to neutron field characterizations at different radiation environments, which is the focus of this paper. DIAMON allows for the measurement of the energy spectra from thermal to fast energies (20 MeV, LE version) or high energies (5 GeV, HE version) without the need of any external input about the guess spectrum, while providing a description of the direction distribution of impinging neutrons. From the knowledge of the spectrum, DIAMON derives field (fluence, fluence fractions) and dosimetric quantities of interest by applying directly the recommended energy-dependent ICRP coefficients (whatever they are).

To experimentally verify the actual potentialities of the overall measurement approach, two different and complex accelerator facilities were selected, namely the CERN-EU high-energy Reference Field (CERF) facility and the Bern medical cyclotron. The former features a high-energy reference mixed field with several radiation types, including neutrons, photons, protons, pions, electrons and muons^34^; the latter is an 18 MeV medical cyclotron used for research activities by the group of the University of Bern, as well as for commercial production of radioisotopes in nuclear medicine^35^. It should be noted that a reference neutron field refers to a fully characterized neutron field, namely with neutron data (fluence, dose, and energy) precisely known. There is a very limited number of reference neutron facilities in the world, including the CERF facility. A DIAMON-HE was used at CERF and the measurement results were compared to the reference data of the facility. Neutron fields were produced at the Bern medical cyclotron by interaction of the 18 MeV proton beam with selected materials. A DIAMON-LE was exposed to these neutron fields and its response was compared to that of the Berthold LB 6411 rem counter and of FLUKA Monte Carlo simulations^36–38^. The next two chapters will present and discuss these results, whereas the description of the DIAMON spectrometer, of the CERF facility and of the Bern medical cyclotron is reported in the “Methods” Section.

## Results

This section reports on the outcomes of two measurement campaigns at two facilities featuring complex radiation environments, namely the CERF facility and the Bern medical cyclotron^35,39^. In particular, we compared the DIAMON-HE and -LE results to that of FLUKA simulations in both the measurement campaigns. The DIAMON-LE response was also compared to that of the Berthold LB 6411 rem counter at the Bern medical cyclotron. Finally the neutron field produced during a routine ^18^F production for nuclear medicine applications was measured with a DIAMON-LE. It should be noted that the CERF facility provides a high-energy reference field for the validation of new detectors, whereas and the Bern medical cyclotron is not characterized in terms of neutron fields.

### Characterization of neutron fields at CERF

**Figure 1:**
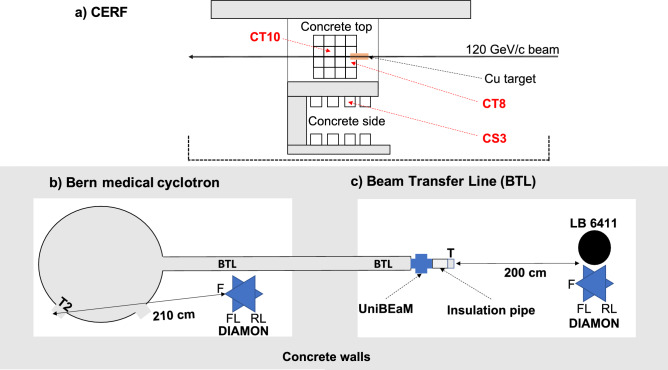
Schematics (top view) of the experimental setup at the CERF (**a**), at the cyclotron bunker (**b**) and at the Beam Transfer Line (BTL) bunker (**c**) of the Bern cyclotron laboratory. The labels “T2” and “T” represent the targets for the neutron production (T2: ^18^O-enriched water target, and “T”: Al or Cu target). The labels “F”, “FL”, and “RL” represent the DIAMON directions “front”, “front-right”, and “rear-left”.

The CERF facility features 40 reference positions for which the composition of the radiation field and of the energy spectrum is precisely known^34^. The reference positions are labelled as IT (Iron Top), CT (Concrete Top) and CS (Concrete Side) followed by a number (e.g. IT1, CT2, CS3, etc.). The aspects of the CERF facility relevant for this work are provided in the “Methods” section, whereas an extensive description is reported in the referred works. A high-energy DIAMON was positioned in the reference positions CT8, CT10 and CS3, which are highlighted in red in Fig. 1a.

**Figure 2:**
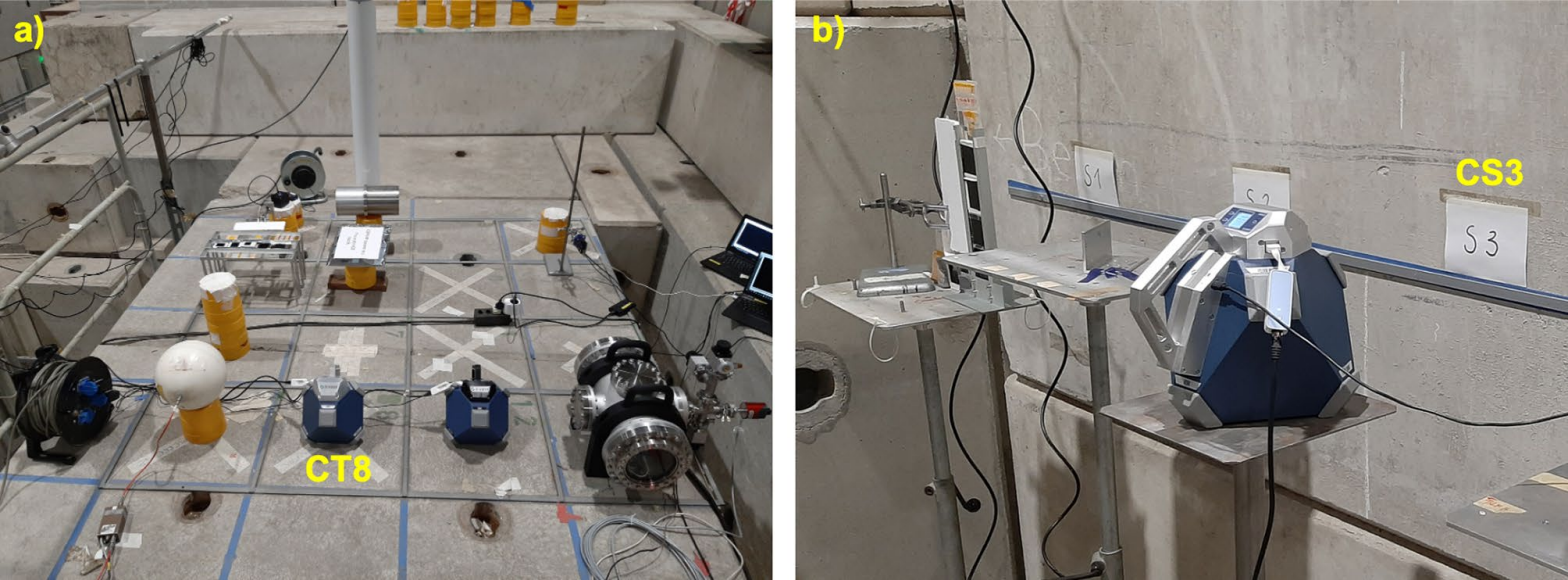
DIAMON-HE in the CERF reference positions CT8 (**a**) and CS3 (**b**).

Figure 2 shows the CERF reference positions on the concrete top-shield and on the concrete side-shield with the DIAMON placed in CT8 (a) and CS3 (b).

**Figure 3:**
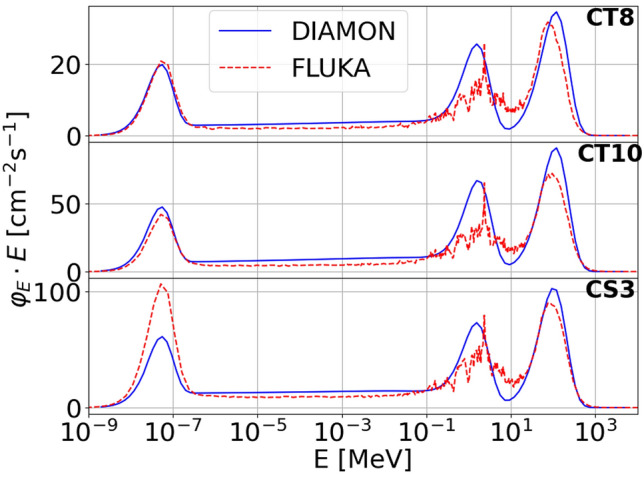
Measured (blue solid line) and FLUKA (red dashed line) neutron spectra in the reference positions CT8 (up), CT10 (middle) and CS3 (bottom) at CERF. The number of primary particles per second, assessed with an ionization chamber was 3.7 $\times 10^6$ s^−1^, 7.8 $\times 10^6$ s^−1^, and 7.9 $\times 10^6$ s^−1^ for CT8, CT10, and CS3, respectively.

Figure 3 shows the measured (blue solid line) and FLUKA (red dashed line) neutron energy spectra for the reference positions CT8, CT10 and CS3. The spectra are plotted in lethargy representation, that is the spectral fluence rate $\Phi _{E}$ multiplied by the central value $E_{c}$ of the energy bin is plotted as a function of the neutron energy. DIAMON neutron spectra output files are directly provided in terms of lethargy, whereas the output of FLUKA simulations is the differential distribution of fluence in energy per incident beam particle (primary particle)^40^. Thus, the FLUKA spectra of figure 3 were scaled by the number of primary particles measured with an ionization chamber used to determine the beam intensity. The measured and simulated neutron energy spectra have the typical shape of neutron spectra in a high-energy field with three pronounced peaks. The first peak from the left, known as thermal peak, is due to neutrons with reduced energy after they scatter on the walls or on any materials in the room and return to the detector location. The peak ranging from approximately 100 keV to 10 MeV is mainly due to nuclear evaporation processes and is known as fast neutron peak. Between these two peaks, the neutron energy spectrum is characterized by a continuous (or epithermal) energy distribution. Finally, the high-energy peak is due to direct neutrons ranging from approximately 10 MeV to the beam energy.

### Characterization of proton-induced neutron fields at the Bern medical cyclotron

**Figure 4:**
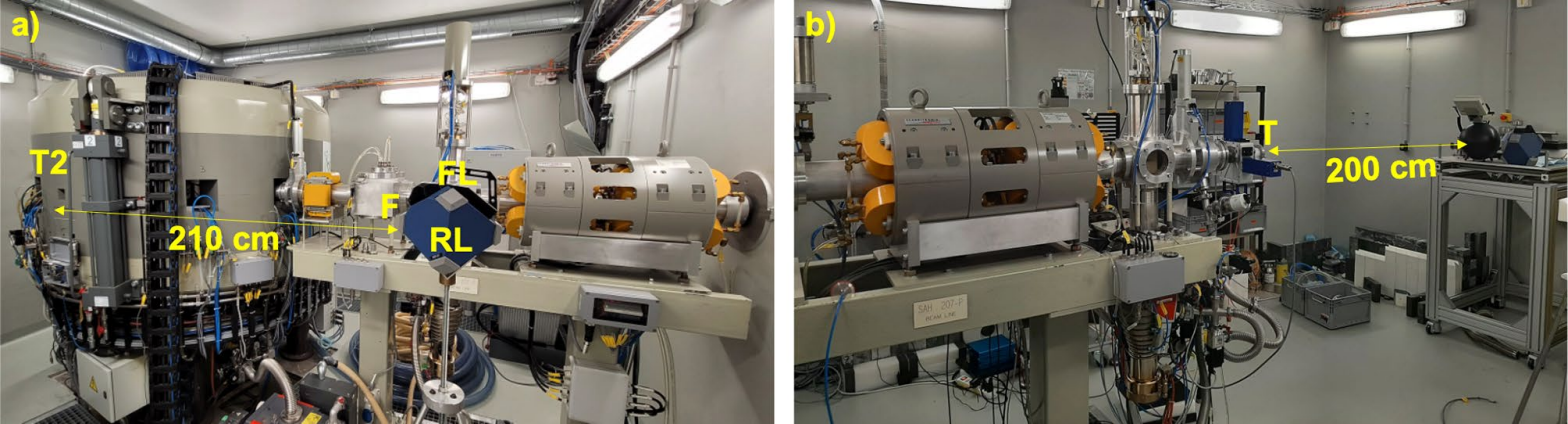
Cyclotron bunker with DIAMON-LE placed on a tripod (**a**). Beam Transfer Line (BTL) bunker with DIAMON-LE and LB 6411 rem counter placed on a table at 2 m distance from the target (**b**). The nomenclature is the same as of Fig. 1.

Neutron beams at the Bern medical cyclotron were produced with a ^18^O-enriched water target, as shown in Figs. 1b and  4a, as well as by interaction of the 18 MeV proton beam with an Al or a Cu target, as shown in Figs. 1c and 4b. Details of the experimental procedure are given in the “Methods” section. The schematic of Fig. 1c shows the DIAMON-LE and the LB 6411 rem counter placed at 200 cm distance from the target (labelled as “T”). The same experimental configuration is also depicted in Fig. 4b.

**Figure 5:**
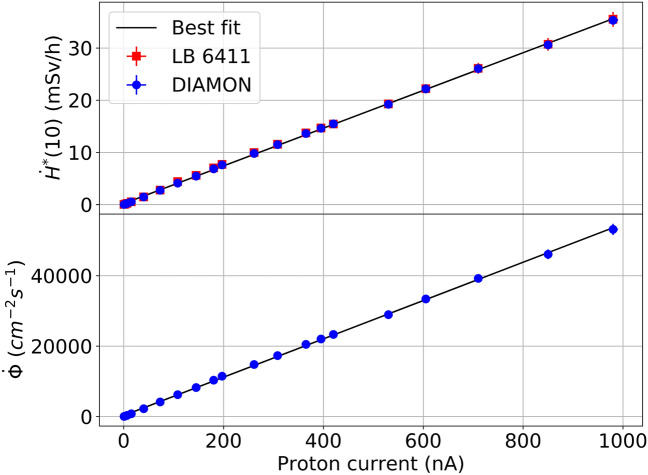
Neutron ambient dose rate of the DIAMON-LE and of the LB 6411 rem counter as a function of the proton beam current (up). DIAMON fluence rate as a function of the proton beam current (down).

We compared the dose response of the DIAMON-LE to that of the LB 6411 rem counter. Figure 5 shows the neutron ambient dose rate $\dot{H}^{*}(10)$ of the DIAMON-LE and of the LB 6411 rem counter as a function of the proton current (up); in addition to the dose response, DIAMON measures also field quantities^33^. Thus, the DIAMON neutron fluence rate $\dot{\Phi }$ is also plotted in Fig. 5 as a function of the proton current (down). This measurement was performed by delivering the 18 MeV proton beam onto the Al target.

**Figure 6:**
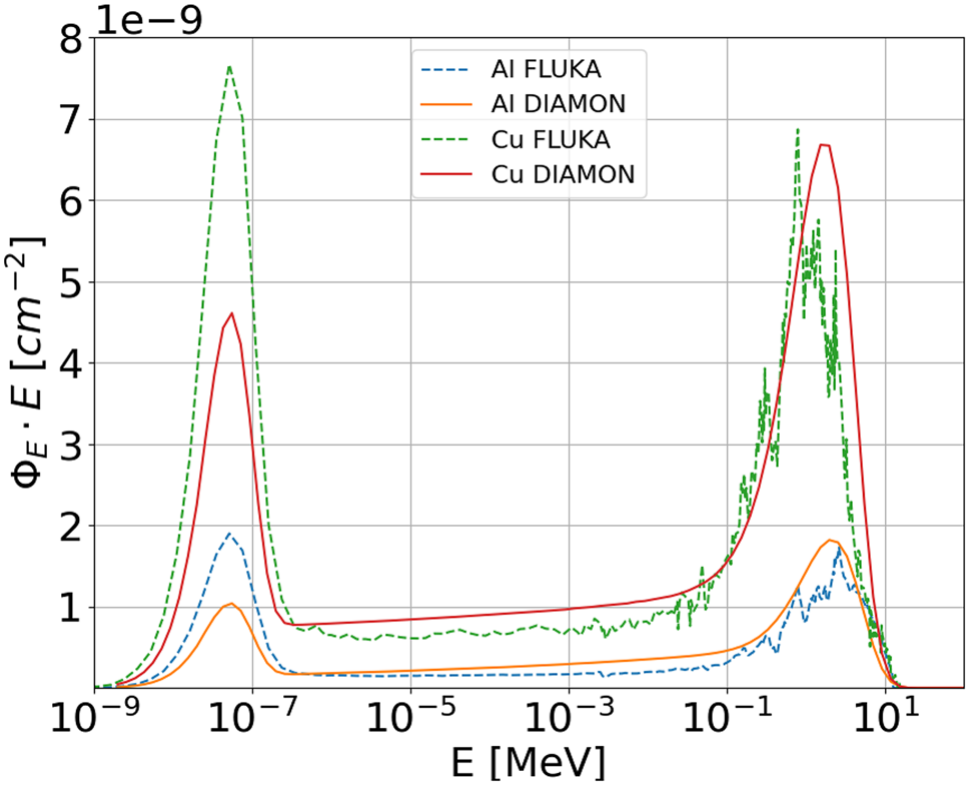
Measured and FLUKA neutron energy spectra produced by interaction of the 18 MeV proton beam with Al and Cu targets. The spectra are normalized per incident primary particle.

**Figure 7:**
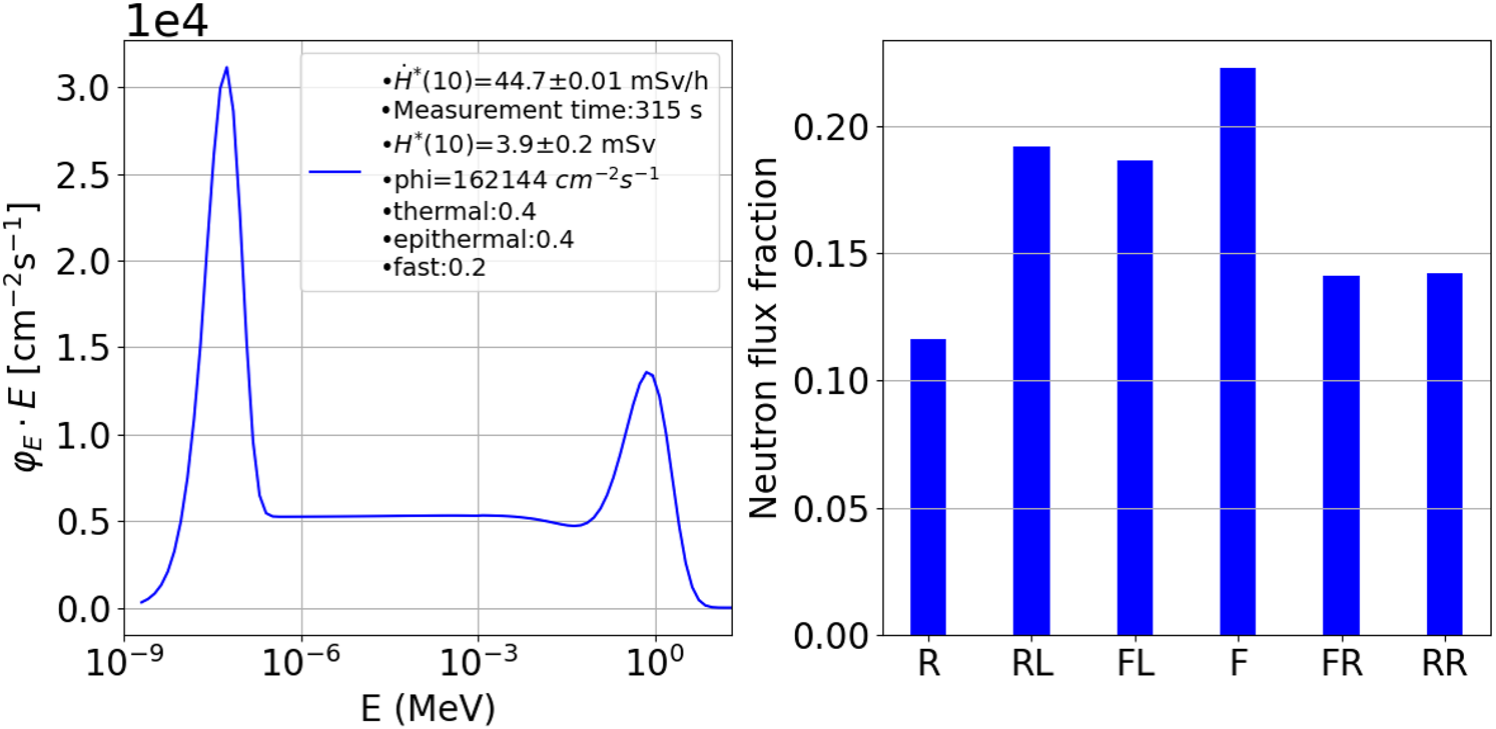
Neutron energy spectrum measured with the DIAMON-LE, resulting from the irradiation of an ^18^O-enriched water target typically used for the ^18^F production for nuclear medicine applications the Bern medical cyclotron (left). Bar chart of the directions distribution of the same neutron field (right). The labels “R”, “RL”, “FL”, “F”, “FR”, and “RR” in the bar char stand for “rear”, “rear-left”, “front-left”, “front”, “front-right”, and “rear-right”, respectively.

Figure 6 shows the measured and FLUKA simulated neutron energy spectra produced by interaction of the 18 MeV proton beam with the Al and Cu targets. The spectra are plotted in units of neutron fluence $\Phi$ normalized to the incident primary particles as a function of the neutron energy (note the difference with respect to the lethargy representation of the results in Fig. 3). As for the high-energy case, the measured and simulated neutron energy spectra have the typical shape with the thermal peak, the fast neutron peak from approximately 100 keV to the beam energy, and the epithermal continuous in between. The measured neutron yields are $(1.04 \pm 0.03) \cdot 10^{-8}$ cm^−2^ and $(3.90 \pm 0.10) \cdot 10^{-8}$ cm^−2^ for Al and Cu targets, respectively.

The measurement of the neutron spectrum produced in a routine ^18^F production for Positron Emission Tomography (PET) applications at the Bern cyclotron laboratory was performed in the experimental configuration depicted in Fig. 1b, and in Fig. 4a with the DIAMON-LE placed on a tripod, 210 cm away from the ^18^O-enriched water target (labelled as “T2”). Figure 7 shows the neutron energy spectrum (left) and the bar chart (right) of the directions of the neutron field resulting from the proton irradiation of the ^18^O-enriched water target. The labels “R”, “RL”, “FL”, “F”, “FR”, and “RR” in the bar char represent six main directions of DIAMON and stand for “rear”, “rear-left”, “front-left”, “front”, “front-right”, and “rear-right”, respectively. Three of these directions (“F”, “FL”, and “RL”) are pointed out in the schematic of Fig. 1b and c, as well as in the picture of Fig. 4a. The data in the bar chart are normalized to the sum of the counts measured along the DIAMON six main directions. DIAMON measures a quasi-isotropic neutron field as a result of the dominant contribution of thermal neutrons (thermal: 0.4 , epithermal: 0.4, fast: 0.2) in the energy spectrum. The larger neutron contribution comes from the forward and left directions (“F”: 22.2%, “RL”: 19.2%, and “FL”: 18.6%) as expected being these faces direct towards the target. The target was irradiated with an average proton current (measured on the target) of ($1.7\pm 0.1)~\upmu A$ for 315 s. The neutron ambient dose rate was measured to be $\dot{H}^{*}(10)=(44.7 \pm 0.01$) mSv/h.

## Discussion

The agreement between DIAMON measurements and FLUKA simulations is satisfactory both for the measurements at the CERF facility (Fig. 3) and at the Bern medical cyclotron (Fig. 6). Since the accuracy of Monte Carlo simulations in reproducing the thermal peak crucially depends on the correct implementation of the geometry of the bunker and its environment, the agreement between measured and simulated spectra is generally worse in this energy range. As for the measurements at CERF, the agreement on the thermal peak for the concrete top-shield reference positions is better than on the concrete side-shield. Results of previous dosimetry measurements at CERF also confirm a worse agreement between measurements and simulations on the concrete-side shield^34^. The evaporation peak and the high-energy peak are well reproduced by FLUKA for all measurements. It is important to note that the good agreement between measurements and simulations is not obvious for the complex mixed radiation fields used in this work. Indeed, the radiation field at the CERF is mostly made of neutrons and gamma rays, with neutrons spanning over a wide energy range from thermal to the beam energy, and gamma rays from about 100 keV. The CERF features also a relevant contribution of electrons and positrons from about 1 MeV, as well as of pions, protons and muons (these latter to a lesser extent). The radiation field at the Bern medical cyclotron produced by interaction of 18 MeV protons with Al, Cu and ^18^O-enriched water targets comprises neutrons and gamma rays. It should also be noted that while the CERF is a well-known reference field, medical cyclotrons are typically not characterized in terms of neutrons. Thus, as a further validation of DIAMON measurements, we compared the DIAMON response to that of the well-established Berthold LB 6411 rem counter. The results of this measurement, reported in Fig. 5a, show that the DIAMON and Berthold LB 6411 $\dot{H}^{*}(10)$ responses are the same within the experimental uncertainties, and they are linear from ($0.070 \pm 0.003$) mSv/h to ($36 \pm 3$) mSv/h. In addition, Fig. 5 shows that DIAMON provides the dose (a), as well as the fluence rate (b). This is a unique feature of DIAMON with respect to other commercial neutron spectrometers.

The thorough knowledge of proton-induced neutron beams at medical cyclotrons is instrumental for several research and industrial activities, including radiation protection, neutron metrology, developments and tests of neutron detectors, activation studies for the decommissioning and neutron damage studies. For example, CaCO_3_ powders have been exposed to neutrons (generated during routine productions of radiopharmaceuticals at the Bern medical cyclotron) with the aim of producing ^37^Ar, a radioisotope of interest for the detection of underground nuclear explosions and for groundwater dating applications^41^. In this work, we also studied neutron fields produced by interaction of 18 MeV proton beams with Al and Cu as target materials. As expected, the neutron yield of Cu is significantly higher than for the Al target. Moreover, we previously measured a negligible contribution of neutrons with a graphite target, which results from the high energy threshold (20.7 MeV) for the ^12^C(p,n)^12^N reaction^42^. Since we aim to establish a scientific methodology for the production of controlled neutron beams with medical cyclotrons, these findings are of paramount importance. In particular, this work is at the basis of the implementation of a neutron beam line at the Bern medical cyclotron to produce different proton-induced neutron beams, by acting on the beam parameters and/or on the target material and geometry. To this end, DIAMON has been found to be a unique instrument in the context of available neutron detectors. Apart from being easy to use and portable, it allows to assess the energy spectrum, field quantities, direction, and the dose of the neutron field in a wide energy range. Furthermore, the excellent agreement between measurements and simulations of the neutron energy spectra measured at two different facilities such as the CERF and the Bern medical cyclotron established that DIAMON can be successfully employed for the characterization of complex and unknown neutron fields.

## Methods

### Direction-aware isotropic and active monitor (DIAMON)

DIAMON is an innovative active neutron spectrometer based on a patented design (Patent: US2020379134). Its polyhedral shape provides an isotropic response while reducing the overall body weight. The spectrometer body is made of polyethylene to moderate impinging neutrons. The thermalized neutrons are detected by a matrix of semiconductor-based sensors, properly distributed at different radial positions along three main orthogonal axes. A low-noise low-power electronics collects signals from sensors and a microprocessor unit manages the data acquisition. A specific software provides two different datasets. The former (“energy outputs”) is obtained by the weighted sum of counts from the sensors located at the same radial position; the latter (“direction outputs”) is given by the weighted sum of counts from the sensors of the same semi-axes. DIAMON is equipped with a proprietary unfolding software, named UNCLE, which implements an internal guess generator, fast convergence criteria and minimization procedures that are specifically designed to operate autonomously. UNCLE exploits a response matrix (numerically assessed through the Monte Carlo code FLUKA and experimentally verified at different monoenergetic neutron fields) to process all the above-mentioned datasets and assess in real-time the energy spectrum and direction of impinging neutrons. Dosimetric quantities of interest are provided as the result of a numerical convolution between the measured energy spectrum and the reference conversion coefficients. Field quantities, such as fluence and field fractions, are also available for the user. The device is available in two different configurations: LE (Low energy) version, suited for neutron field measurements ranging from thermal up to 20 MeV, and HE (high energy) version, which extends the spectrometer response to higher energies. In this latter case, the main moderator body is also equipped with high-Z inserts. The mechanical design is especially conceived to have a light device (about 6 kg for LE version and 8.5 kg for HE version).

### The CERN-EU high-energy reference field (CERF)

The CERF is installed in the North Experimental Area on the Prévessin site of the European Organization for Nuclear Research (CERN). It provides a reference mixed radiation field for the calibration and test of instrumentation used at high-energy accelerators, as well as for aircraft and space dosimetry^43–45^. The radiation field generated at the CERF originates from a secondary beam from the Super Proton Synchrotron (SPS). The interaction of this secondary beam, made of positive pions (57%), protons (39.7%) and positive kaons (3.3%) with a momentum of 120 GeV/c, on a 50 cm length and 7 cm diameter Cu target produces the CERF mixed radiation field^46^. The Cu target can positioned below either an 80 cm thick concrete roof-shield or a 40 cm thick iron roof-shield. A total of 40 reference positions, for which either the composition and the energy spectra of the radiation field are precisely known (by means of FLUKA simulations), is available.

In our measurements, the Cu target was placed below the 80 cm thick concrete roof-shield. In particular, we measured the neutron energy spectra with the DIAMON-HE placed in the reference positions CT8, CT10, and CS3, in the experimental configuration shown in Fig. 1a, and in the pictures of Fig. 2. The beam monitoring on which the normalization of the FLUKA reference spectra relies, was performed by means of an air-filled, parallel-plate, transmission-type ionization chamber positioned just before the Cu target^34^. The measured number of primary particles per second is 3.7 $\times 10^6$ s^−1^, 7.8 $\times 10^6$ s^−1^, and 7.9 $\times 10^6$ s^−1^ for the measurement runs in CT8, CT10, and CS3, respectively.

### The Bern cyclotron laboratory

The Bern cyclotron laboratory is located at the Bern University Hospital (Inselspital). It hosts an 18/18 IBA Cyclone, accelerating H^−^ ions to a nominal energy of 18 MeV with a variable beam current from a few pA to 150 μA^47–49^. The cyclotron is used overnight for the commercial ^18^F production necessary for the synthesis of fluorodeoxyglucose for the Positron Emission Tomography (PET) imaging; during the day, multi-disciplinary research activities are carried out by the Laboratory for High Energy Physics (LHEP) of the University of Bern. These activities include the production of novel radioisotope for theranostics, developments on particle accelerators and detectors for medical applications, as well as radiation hardness^50–54^. The cyclotron features eight independent exit ports. Four of them (plus two spare) are connected to ^18^O-enriched water targets for routine ^18^F production. One exit port has been recently equipped with a Mini-PET Beamline (MBL) for the production of non-conventional radioisotopes for theranostics; the last exit port is connected to a 6 meter long Beam Transfer Line (BTL)^55–57^. The BTL, ending in a second bunker with independent access to the experimental area, is equipped with beam focusing and diagnostics systems including a two-dimensional beam monitor based on scintillation optical fibers, named UniBEaM, developed by LHEP and commercialized by the Canadian company D-Pace^49,58^. The schematics and pictures of the cyclotron bunker and of the BTL bunker at the Bern cyclotron laboratory are reported in Figs. 1 and 4, respectively.

Neutron beams have been produced by interaction of a proton beam of nominal energy 18 MeV with an Al and a Cu target. Both the targets are shaped as KF-40 blank flanges of thickness greater than the proton range of 18 MeV, so that they fully stop inside the targets. The two targets were mounted at the end of the BTL and were electrically isolated with respect to the rest of the beam pipe by means of a 10 cm long polymethyl methacrylate (PMMA) insulation pipe (Fig. 1c). The targets were electrically connected to a Keysight B2985A Picoammeter, connected to a PC by means of a custom LabView graphical user interface, which provides the beam current as a function of time^59^. The beam profiles measured with the UniBEaM detector, positioned 13 cm downstream the target, had a Full Width at Half Maximum (FWHM) of less than 3.5 mm (for both cases) and were centered in the beam pipe, implying an optimal focusing of the proton beam on the targets. For the measurements of the neutron energy spectra with Al and Cu targets, DIAMON-LE was positioned at 200 cm distance from the target along the direction of the beam pipe central axis (as shown in Figs. 1c and 4b). Also for the measurements aiming at the comparison of the responses of the DIAMON-LE and of the LB 6411 rem counter, we placed both instruments in this position. The Al and Cu target were exposed to the proton beam for 174 s and 294 s with an average proton current of 335 nA and 246 nA, respectively. Finally, to study the neutron field produced during a standard ^18^F production we fixed a DIAMON-LE on a tripod in the cyclotron bunker, as shown in Figs. 1b and 4a. For this measurement, the exit port number 2 (labelled as “T2” in Fig. 4) was filled with a ^18^O-enriched water target. After preliminary beam focusing and centering, we irradiated the ^18^O-enriched water target for 315 s with an average beam current (measured on the target) of (1.8 ± 0.1) μA.

### FLUKA Monte Carlo simulations

In order to assess the DIAMON measurements at the CERF we used the reference energy spectra provided by the facility, which are based on FLUKA Monte Carlo simulations. FLUKA simulations for the experimental set-up with the Cu target under the concrete roof-shield at CERF were performed with the FLUKA2011 version 2x.2 (May 2018), and with ”PRECISIOn” settings. As the primary beam at CERF is a mixed field, the FLUKA reference energy spectra result from three simulation runs performed for each particle type, according to their statistical weight (57.0% for positive pions, 39.7% for protons and 3.3% for positive kaons)^34^.

The Bern medical cyclotron experimental setup was simulated with FLUKA version 4.0 and Flair 3.1 (2020)^36–38,60^. With a beam energy of $18 \, \mathrm {MeV}$ there is no point-wise transport of neutrons (and no nuclear model) for the materials in our simulation. The neutrons are binned into 260 energy groups and transported according to specific transition probabilities^40^. Apart from a mild biasing inside the targets to increase the number of produced neutrons, we did not change any simulation parameters and relied on the default “PRECISIOn” settings. We used the technical drawings of the target and of the cyclotron bunker for the implementation geometry. Particular care was given to the geometry of the target, since this significantly impacts on the shape of the neutron spectrum around the dipole resonance. Obviously, without the bunker walls there would be no thermal peak in the neutron spectrum. However, we noticed that the neutron spectrum exhibits only minor changes in the thermal peak when additional objects of different materials, such as beam line magnets, tables, or other equipment, are included in our simulation. Also variations of the beam parameters have not shown a strong impact on the neutron spectrum. The beam energy extracted to the BTL was accurately measured by previous experiments^51,61–63^. Thus, we used the measured energy value of ($18.2 \pm 0.4)\, \mathrm {MeV}$ energy with a circular Gaussian shape of $3.5 \, \mathrm {mm}$ FWHM. The double differential fluence as well as the effective dose were scored within an air-filled sphere with $r=5 \, \mathrm {cm}$ at the detector location. As usual, all quantities that are scored in the simulation are normalized to the number of primary particles. It is therefore of particular importance to have a reliable measurement of the beam current in order to have the correct normalization when comparing with the DIAMON’s measurements.

## Data Availability

The datasets generated during and/or analysed during the current study are available from the corresponding author on reasonable request.
